# A pan-kidney cancer study identifies subtype specific perturbations on pathways with potential drivers in renal cell carcinoma

**DOI:** 10.1186/s12920-020-00827-5

**Published:** 2020-12-28

**Authors:** Xiaohui Zhan, Yusong Liu, Christina Y. Yu, Tian-Fu Wang, Jie Zhang, Dong Ni, Kun Huang

**Affiliations:** 1 National-Regional Key Technology Engineering Laboratory for Medical Ultrasound, Guangdong Key Laboratory for Biomedical Measurements and Ultrasound Imaging, School of Biomedical Engineering, Health Science Center, Shenzhen University, Shenzhen, 518037 China; 2 Department of Medicine, Indiana University School of Medicine, Indianapolis, IN 46202 USA; 3 School of Basic Medicine, Chongqing Medical University, Chongqing, 400016 China; 4 College of Automation, Harbin Engineering University, Harbin, 150001 Heilongjiang China; 5 Department of Biomedical Informatics, The Ohio State University, Columbus, OH 43210 USA; 6 Department of Medical and Molecular Genetics, Indiana University School of Medicine, Indianapolis, IN 46202 USA; 7 Regenstrief Institute, Indianapolis, 46202 USA

**Keywords:** RCC, ccRCC, PRCC, ChRCC, Pathway perturbation, Upstream regulator, Prognostic pathway

## Abstract

**Background:**

Renal cell carcinoma (RCC) is a complex disease and is comprised of several histological subtypes, the most frequent of which are clear cell renal cell carcinoma (ccRCC), papillary renal cell carcinoma (PRCC) and chromophobe renal cell carcinoma (ChRCC). While lots of studies have been performed to investigate the molecular characterizations of different subtypes of RCC, our knowledge regarding the underlying mechanisms are still incomplete. As molecular alterations are eventually reflected on the pathway level to execute certain biological functions, characterizing the pathway perturbations is crucial for understanding tumorigenesis and development of RCC.

**Methods:**

In this study, we investigated the pathway perturbations of various RCC subtype against normal tissue based on differential expressed genes within a certain pathway. We explored the potential upstream regulators of subtype-specific pathways with Ingenuity Pathway Analysis (IPA). We also evaluated the relationships between subtype-specific pathways and clinical outcome with survival analysis.

**Results:**

In this study, we carried out a pathway-based analysis to explore the mechanisms of various RCC subtypes with TCGA RNA-seq data. Both commonly altered pathways and subtype-specific pathways were detected. To identify the distinctive characteristics of each subtype, we focused on subtype-specific perturbed pathways. Specifically, we observed that some of the altered pathways were regulated by several recurrent upstream regulators which presenting different expression patterns among distinct RCC subtypes. We also noticed that a large number of perturbed pathways were controlled by the subtype-specific upstream regulators. Moreover, we also evaluated the relationships between perturbed pathways and clinical outcome. Prognostic pathways were identified and their roles in tumor development and progression were inferred.

**Conclusions:**

In summary, we evaluated the relationships among pathway perturbations, upstream regulators and clinical outcome for differential subtypes in RCC. We hypothesized that the alterations of common upstream regulators as well as subtype-specific upstream regulators work together to affect the downstream pathway perturbations and drive cancer initialization and prognosis. Our findings not only increase our understanding of the mechanisms of various RCC subtypes, but also provide targets for personalized therapeutic intervention.

## Background

Kidney cancer or renal cell carcinoma (RCC) is comprised of distinct histological subtypes which present different genetic characteristics, biological functions and clinical outcome [1]. RCC contains three major histologic subtypes: clear cell RCC (ccRCC), papillary RCC (PRCC) and chromophobe RCC (ChRCC). ccRCC is the most common RCC subtype occupying approximately 75% of RCC cases; PRCC accounts for about 15% and ChRCC represents about 5% of RCC cases [1]. Thus, the treatment of RCC is quite complicated due to the distinct molecular alterations, biological functions and clinical outcome. It is of great interest to explore the molecular basis as well as therapeutic targets for different subtypes of RCC in order to enhance our understanding of different subtypes of RCC and provides new insights for targeted therapy.

Genomic alterations lead to differential expression of genes and subsequently induce dysregulation of biological functions to promote tumorigenesis and development. To date, numerous studies have been carried out to investigate the molecular basis of each subtype and elucidate potential mechanisms of tumorigenesis [1–7]. The Cancer Genome Atlas (TCGA) Research Network has performed a series of studies to investigate the molecular characterizations and altered pathways within different subtypes of RCC [2, 3, 7]. Ricketts et al. [1] conducted a comprehensive analysis to compare the homogeneity and heterogeneity of molecular basis and perturbed pathways among distinct subtypes of RCC. Banumathy et al. [6] summarized the known molecular alterations that lead to perturbations in signaling pathways. Furthermore, the metabolic basis of kidney cancer has also been investigated [8–13]. Despite these remarkable discoveries, the understanding about the mechanisms of various RCC subtypes are still incomplete and further comparative studies are still desired. As genomic alterations are eventually manifested to biological functions, pathway-based analysis can be a good choice to investigate the mechanisms of different subtypes of RCC, which greatly reduces complexity and increases interpretability [10, 12, 13].

In this study, we systematically investigated the mechanisms of various subtypes of RCC at the pathway level, identifying pathway perturbations as well as the potential upstream regulators and exploring the associations with clinical outcome. Previously, Ricketts et al. [1] re-evaluated the histologic subtypes of TCGA kidney cancer samples. With the re-classified histologic subtype samples, we first analyzed the gene expression of each RCC subtype against normal tissues and filtered differentially expressed genes (DEGs) within pathways for further analysis. Considering that genes associated with multiple pathways can be a confounding factor to investigate the pathway perturbations with a specific function, correction analysis was performed for these promiscuous genes across linked pathways. Enrichment analysis was subsequently carried out based on promiscuity-corrected DEGs and significantly disturbed pathways versus normal tissues were obtained for each subtype, including both commonly altered pathways and subtype-specific disturbed pathways among different subtypes of RCC. Specifically, to identify the distinctive characteristics of each subtype, we focused our further analysis on subtype-specific perturbed pathways for both the upstream regulator analysis and survival analysis. It was shown that some of the recurrent upstream regulators were shared among distinct RCC subtypes, with different expression patterns. Additionally, a large number of perturbed pathways were regulated by subtype-specific upstream regulators. Furthermore, we examined the relationships between subtype specific perturbed pathways and cancer patient outcome (i.e., survival time). Prognostic pathways were identified for each subtype and their roles in tumor development and progression were inferred. In summary, our work explored the relationships among pathway alterations, upstream regulators as well as clinical outcome for different subtypes of RCC. We hypothesized that various upstream regulators together affect the downstream pathway perturbations that drive the tumor procession. Our findings provide new insights for tumorigenesis and development of various RCC subtypes and provide promising targets for precision therapy.

## Methods

### Experimental design and statistical rationale

**Fig. 1:**
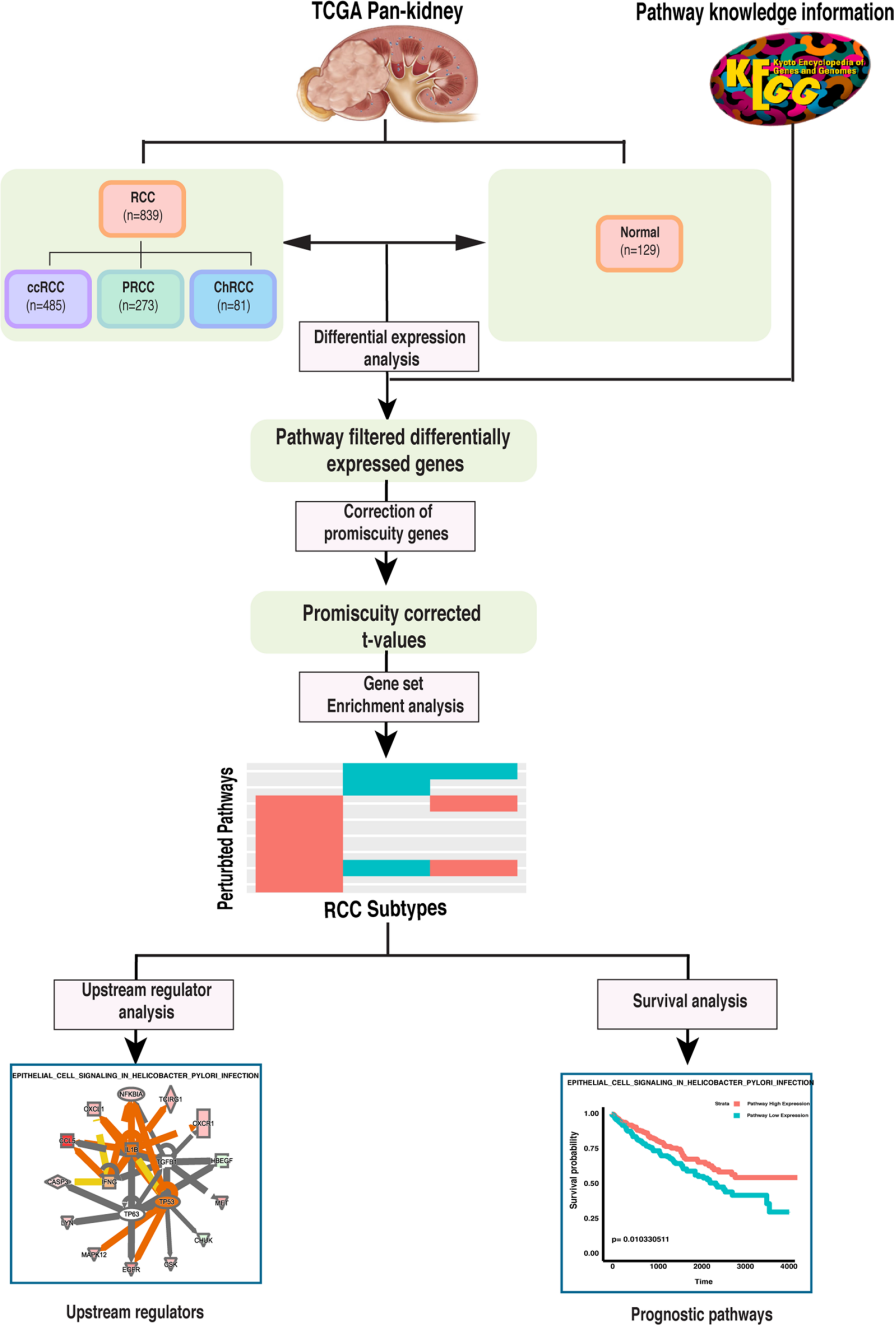
Study workflow: The workflow describes the method of pathway-based analysis to identify the pathway perturbations, the potential upstream regulators and prognostic pathways of various RCC subtypes. Based on re-evaluated RCC subtype samples, differential gene expression analysis was performed for each subtype against normal tissue; Differentially expressed genes (DEGs) within pathways (KEGG) were selected for further analysis for each subtype; Wald t-values for promiscuous-genes associating with multiple pathways was corrected; Enrichment analysis was performed based on corrected t-value of DEGs within pathways, and significantly altered pathways were identified for each subtype; Upstream regulators analysis was performed to explore the potential drivers of each subtype-specific pathway; survival analysis was carried out to evaluate the relationship between subtype-specific pathway and patient clinical outcome for each subtype

The main objective of this study was to investigate the pathway perturbations of various RCC subtypes against normal tissue, identify potential upstream regulators and explore the relationships associated with clinical outcome. The whole workflow was shown in Fig. 1. Firstly, we performed differential gene expression analysis based on re-evaluated RCC subtype samples and filtered the differentially expressed genes (DEGs) within pathway of each subtype. Secondly, we corrected the Wald t-value obtained from DESeq2 analysis for promiscuous genes that participated in multiple pathways. Thirdly, we carried out the enrichment analysis based on the corrected-t value of DEGs and acquired the significantly altered pathways of each subtype. Fourthly, we compared the pathway perturbations among various RCC subtypes. Then we identified the potential upstream regulators of subtype specific pathways with Ingenuity Pathway Analysis (IPA) and summarized the recurrent upstream regulators underlying various disturbed pathways. Finally, we explored the relationships among perturbed pathways, patient outcome, and the underlying upstream regulators.

### Data sources and pre-processing

The RNAseqV2 level 3 data including raw counts data, normalized scaled estimate data, and corresponding clinical data for TCGA Pan-kidney cohort were accessed from the Broad GDAC Firehose (https://gdac.broadinstitute.org). The histological subtype of each sample were re-divided based on the original pathology reports or re-evaluated by urologic pathologists in Ricketts et al. [1]. The reclassified TCGA kidney cancer samples contain 839 RCC samples: 485 clear cell RCC (ccRCC), 273 papillary RCC (PRCC), 81 chromophobe RCC (ChRCC), and 129 matched normal samples were used in our analysis. The KEGG pathway knowledge information was downloaded from MSigDB database (http://software.broadinstitute.org/gsea/downloads.jsp) and included 5266 genes and 186 pathways. Demographic and clinical characteristics of the patients are described in Table 1.

**Table 1:** Demographic and clinical characteristics of RCC patients by subtype

Characteristics	ccRCC	PRCC	ChRCC
Patient No.	485	273	81
Age (years)^a^			
Range	26 ~ 90	28 ~ 88	17 ~ 86
Median	61	61	51
AJCC Stage^a^			
Stage I	241	163	28
Stage II	49	21	28
Stage III	118	49	17
Stage IV	77	15	8
Follow-up (days)^a^			
Range	0 ~ 4537	0 ~ 5925	30 ~ 4676
Median	1200	750.5	1731
Vital_Status			
Living	325	233	65
Deceased	160	40	16

^a^Denotes some patients have missing information in these categories

### Differential gene expression and pathway enrichment analysis

To obtain high-quality RNAseq data, raw counts data with a value of zero in more than 20% of the samples in each subtype were excluded from analyses. To obtain DEGs, raw RNAseq counts data of each subtype and normal samples were used to perform the differential expression analysis based on negative binomial generalized linear model with R package DESeq2 (version 1.24.0) [14]. In particular, for the differential expression analysis in DESeq2, significance testing with Wald test, and multiple testing with Benjamini-Hochberg Procedure were used. Only genes with both Benjamini-Hochberg adjusted q-values < 0.05 and |fold change| > 1.5 were considered as DEGs. In addition, pathway DEGs were further filtered based on KEGG pathway knowledge information. DEGs were kept only if they were present in KEGG. The Wald t-value statistics for DEGs resulting from DESeq2 analysis was chosen for subsequent analysis, with a higher t-value associated with a higher statistical significance of differential expression.

As genes associated with multiple pathways can be a confounding factor when linking pathway alternations to a specific function, such kind of genes were subjected to a promiscuity correction [15]. Here, we adopted the same procedure as in Gaude et.al [15] to correct the Ward t-value for differential gene expression of promiscuous-gene across pathways with the following calculation:

$$\mathrm{corrected}\ \mathrm{Ward}\ \mathrm{t}\hbox{-} \mathrm{value}=\mathrm{Wald}\ \mathrm{t}\hbox{-} \mathrm{value}/\mathrm{associated}\ \mathrm{pathway}\ \mathrm{number}.$$

Furthermore, pathway enrichment analyses were also performed based on corrected Wald t-values of DEGs using the R package ‘piano’ (version 2.0.2) [16]. Here, the ‘gsea’ method applying the Gene set enrichment analysis (GSEA) method was used, and significantly up-regulated and down-regulated pathway terms were identified with *p*-value less than 0.05.

### Upstream regulator analysis by ingenuity pathway analysis (IPA)

To identify the potential upstream regulators of a particular pathway, we used the Ingenuity Pathway Analysis (IPA) software and performed the “Core” and “Upstream Regulator” analyses on IPA [17] based on DEGs within each given pathway. The potential upstream regulators were firstly identified based on experimentally observed interactions between upstream regulators and their target genes in human species which have been manually curated in IPA’s Knowledge Base and then further selected using the following criteria: (1) The predicted upstream regulators belong to transcription regulators (TFs), cytokines or growth factors; (2) The predicted upstream regulators showed statistical significance of Fisher’s Exact Test with *p*-value < 0.01; (3) The predicted upstream regulators regulated more than 10% DEGs of a pathway in order to identify the drivers most likely affecting this pathway; (4) The predicted upstream regulator itself was also a DEG.

### Survival analysis

To assess the association between subtype-specific enriched pathways and patient survival information, overall survival (OS) analysis was performed based on pathway expression within each subtype. Here, we calculated the pathway expression based on the DEGs within each pathway. TCGA normalized scaled estimate data were firstly converted into TPM (multiplied by 1e6) value and then subjected to log2(TPM + 1) transformation for subsequent analysis. The expression of each pathway was measured by the average expression of all DEGs inside the pathway based on log2-transformed values. For each subtype-specific enriched pathway, the patient cohort for the corresponding subtype was divided into two groups (i.e. pathway high expression group and pathway low expression group) by average expression of the pathway. Then the Kaplan-Meier estimator was used for patient stratification and the log-rank test was applied to compare the survival difference between two groups with R package ‘survminer’ [18]. A multiple testing correction with Benjamini-Hochberg false discovery rate (B&H FDR) was performed based on log-rank test *p* values across each subtype and pathways with *p*-values less than 0.05 were considered as prognostic pathways.

### Statistical analysis software

Except where noted above, all statistical analyses were performed in R version 3.5.1.

## Results

### Pathway perturbations among various RCC subtypes

**Fig. 2:**
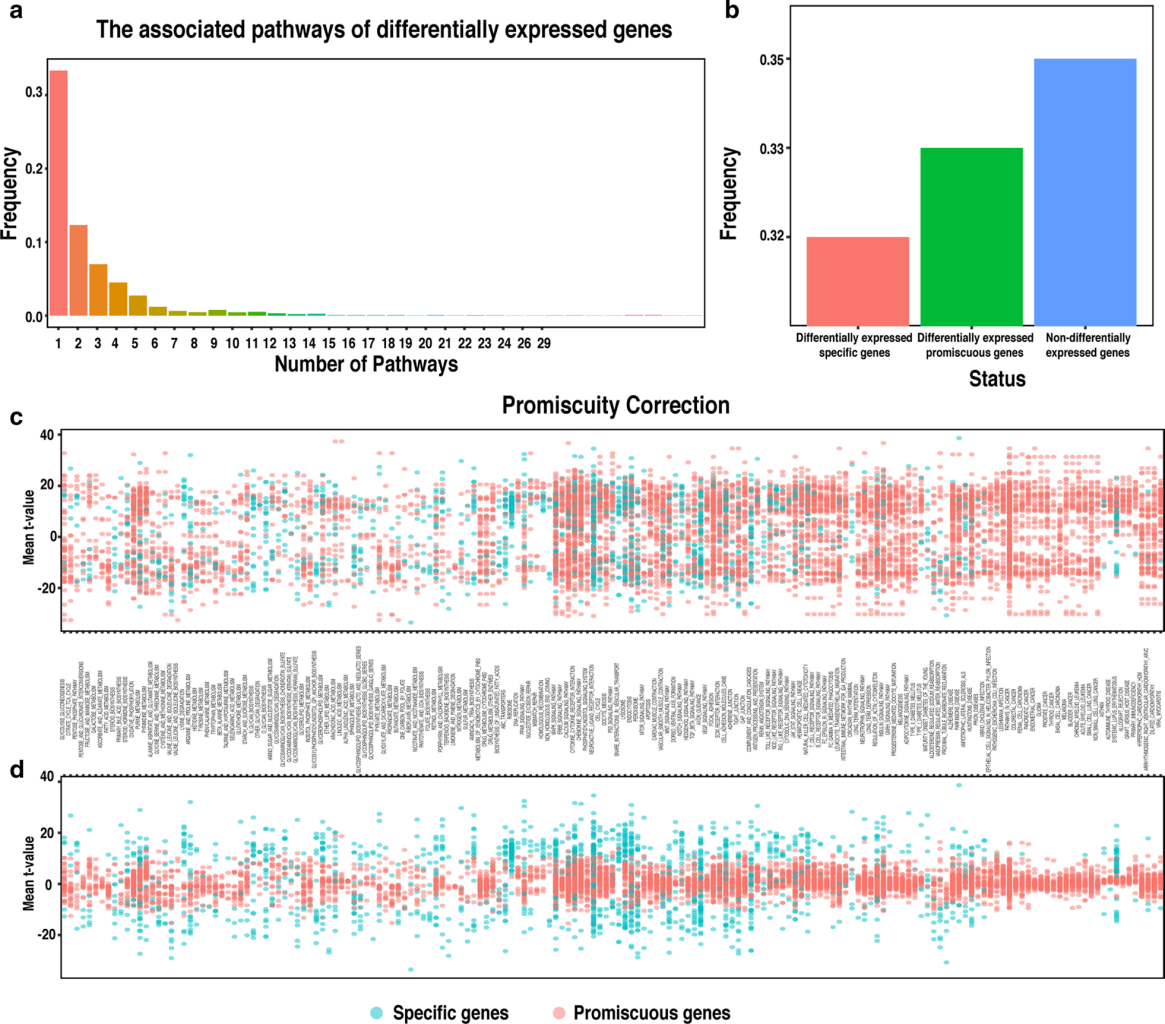
Promiscuity correction of differentially expressed genes across the pathways. **a** The percentage of differentially expressed genes (DEGs) associated with various pathways in all DEGs. X-axis denotes the number of pathways. Y-axis denotes the percentage of DEGs linking with certain pathways in all DEGs. **b** The percentage of DEGs and non-DEGs in pathway genes. The red bar indicates the percentage of DEGs relating to a specific pathway in pathway genes. The blue bar indicates the percentage of non-DEGs in pathway genes. The green bar indicates the percentage of DEGs participating in multiple pathways in pathway genes, which were then subjected to promiscuity correction. **c** Non corrected and **d** promiscuity-corrected t-values of DEGs within certain pathways. Each column represents a pathway and each dot represents a gene. The value of each dot represents the **c** Non corrected and **d** promiscuity-corrected mean t-values of cancer vs normal for pathway genes across RCC subtypes. Specific genes (blue) of a certain pathway and promiscuous genes (red) associating with multiple pathways are highlighted in order to compare the effect of promiscuity correction

**Fig. 3:**
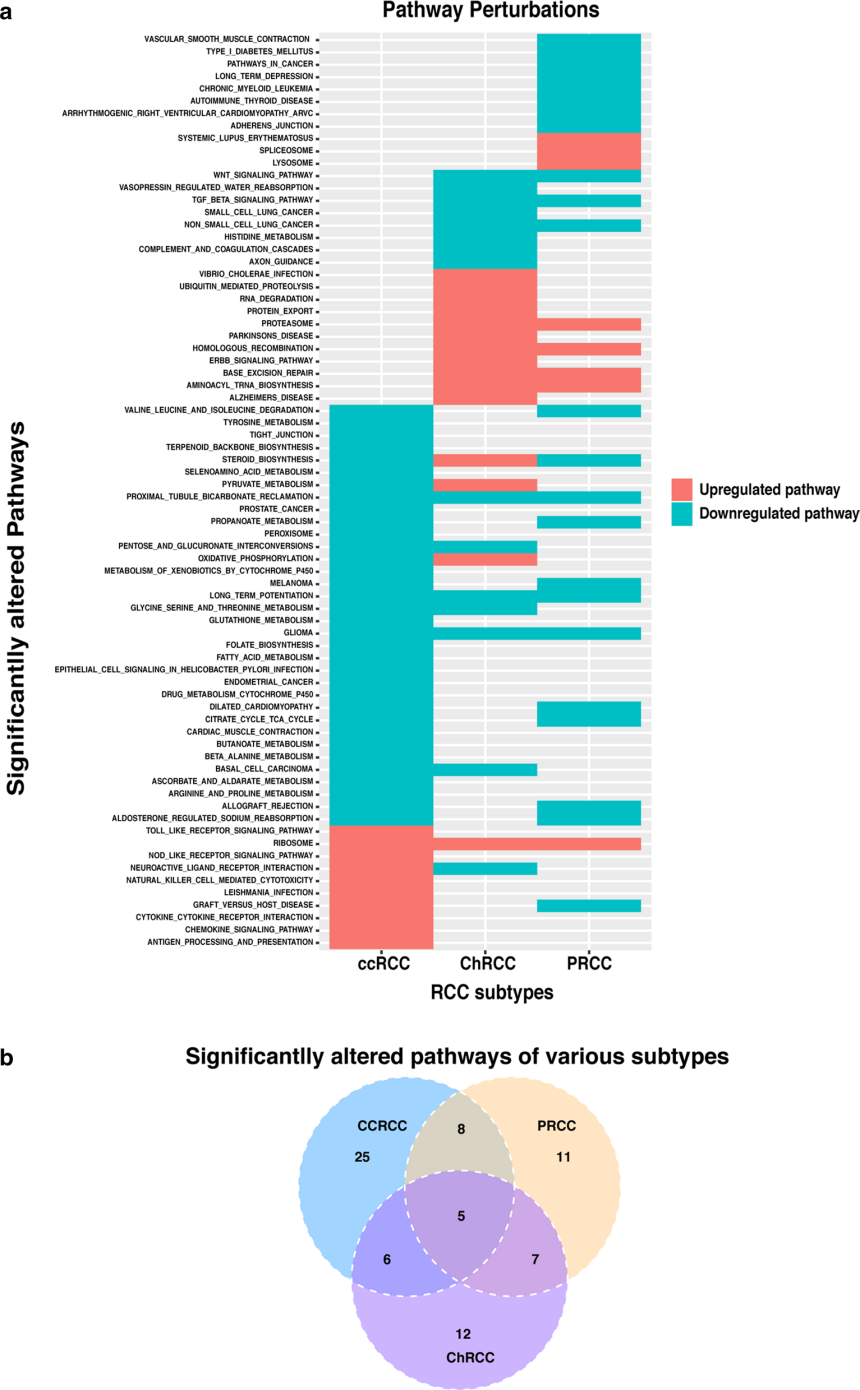
Significantly perturbed pathways for different subtypes of RCC. Heatmap representation of the significantly altered pathways for three subtypes of RCC compared to normal tissues. X-axis lists the distinct subtype of RCC, and y-axis lists significantly altered pathways. Red and blue boxes indicate upregulated and downregulated pathways in different subtypes of RCC compared to normal tissues, respectively. **b** The Venn diagram for the overlap of significantly altered pathways among ccRCC, PRCC and ChRCC

To investigate the pathway perturbations of various RCC subtypes against normal tissues, we analyzed the expression of pathway genes of kidney cancer from TCGA, encompassing 485 ccRCC, 273 PRCC, 81 ChRCC and 129 normal samples (see Fig. 1 for the overall workflow). Differential expression analysis was performed and differentially expressed genes (DEGs) within pathways were selected for the further analysis, including 2198 DEGs for ccRCC, 2129 DEGs for PRCC, and 2243 DEGs for ChRCC. These DEGs mapped to 186 pathways from the KEGG database. After examining these DEGs, we noticed that about 33% DEGs were related to multiple pathways (Fig. 2a, b). As the genes associated with multiple pathways can be a confounding factor, in order to reduce the chance of significantly altered pathways driven by the changes of promiscuous genes without the changes of pathway-specific genes, promiscuity correction analysis was performed on the DEGs associated with multiple pathways (Fig. 2c, d, Additional file 1: Table S1). To identify the pathway perturbations of various subtypes, gene set enrichment analysis was carried out based on the promiscuity-corrected DEGs between each RCC subtype and normal tissues. Significantly dysregulated pathways were identified in each subtype, with some pathways overlapping (Fig. 3a, b, Additional file 2: Table S2). For ccRCC, 44 significantly perturbed pathways were identified, comprised of 10 upregulated pathways and 34 downregulated pathways. For PRCC, 31 significantly altered pathways were identified, comprised of 8 upregulated pathways and 23 downregulated pathways. For ChRCC, 30 significantly disturbed pathways were identified, comprised of 15 upregulated pathways and 15 downregulated pathways.

### Comparison of altered pathways among various RCC subtypes

We compared the significantly altered pathways of each RCC subtype against normal tissues. We noticed that most of the perturbed pathways for ccRCC were associated with immune- and metabolism-related pathways. While for PRCC and ChRCC, they were mainly associated with immune- and blood vessel-related pathways, and mRNA- and protein-synthesis related pathways, respectively. After examining the active pathways among various subtypes, both commonly perturbed pathways for RCC and subtype-specific perturbed pathways were achieved (Fig. 3a, Additional file 2: Table S2). There were five commonly altered pathways among the three subtypes of RCCs, including one consistently upregulated pathway, three consistently downregulated pathways, and one dysregulated pathway (Fig. 3a, Additional file 3: Table S3). Moreover, we observed that over 30% DEGs were overlapped among various subtypes of RCC for the commonly altered pathway (Additional file 4: Table S4). As for the subtype-specific altered pathways, there were 25 dysregulated pathways for ccRCC (7 upregulated and 18 downregulated), 11 dysregulated pathways for PRCC (3 upregulated and 8 downregulated), and 12 pathways for ChRCC (7 upregulated and 5 downregulated) (Additional file 3: Table S3). To identify distinctive characteristics of each subtype, we further analyzed subtype-specific perturbed pathways.

### The potential upstream regulators of perturbed pathways

**Fig. 4:**
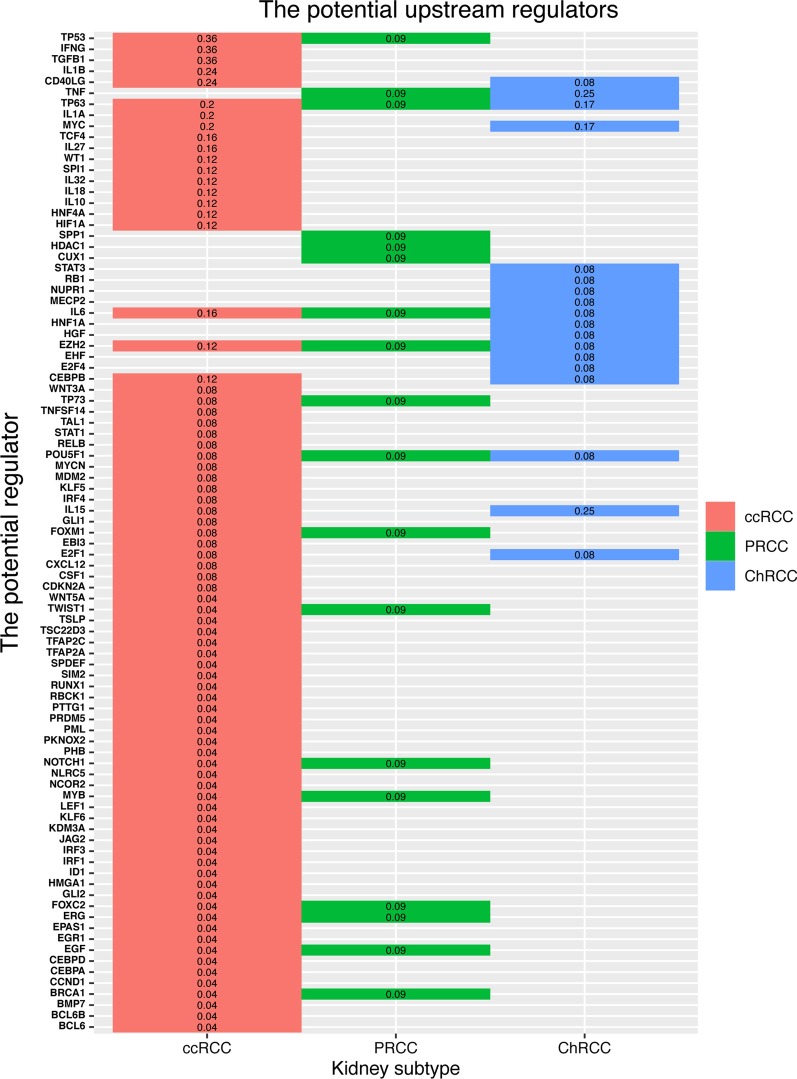
Potential upstream regulators of altered pathways in RCC subtypes. Heatmap representation of the upstream regulators across various RCC subtypes. The values in each box indicate the percentage of downstream altered pathways affected by the particular upstream regulator in each subtype

**Fig. 5:**
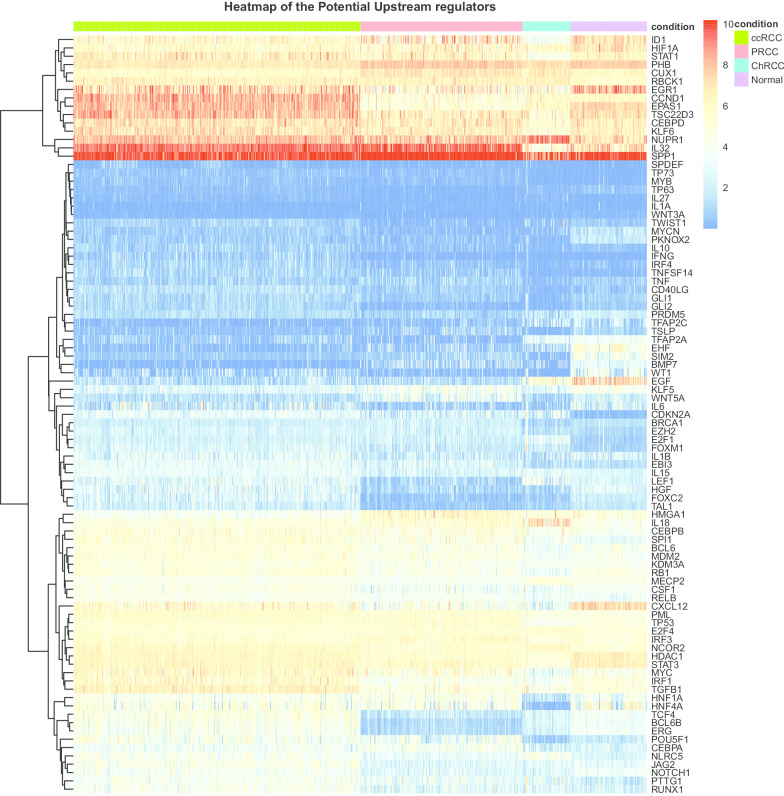
Heatmap of the expression patterns of the potential upstream regulators among various subtypes

Perturbed pathways involve several differentially expressed genes, which work together to activate abnormal biological processes or dysregulate certain biological functions. To systematically explore how changes of pathway components translated into the abnormal activation of certain pathways during tumorigenesis, we performed IPA (Qiagen) Upstream Regulator Analysis based on DEGs within a pathway and the potential upstream regulators for each subtype-specific altered pathway were identified (Additional file 5: Table S5). Note that these regulators regulated over 10% DEGs in a certain pathway. An examples of the correlations between upstream regulators with the target gene is shown in Additional file 1: Fig. S1. A more comprehensive analysis of the potential upstream regulators indicated that some of the significantly altered pathways were regulated by several recurrent upstream regulators, including *TP53, IFNG, TGFB1, TNF*, *TP63* and so on (Fig. 4). Moreover, a few of recurrent upstream regulators were shared among different subtypes of RCC. *TP63* was an important regulator which affected multiple perturbed pathways among three RCC subtypes (regulated 20, 9 and 17% significantly altered pathways of ccRCC, PRCC and ChRCC, separately) (Fig. 4). *TP53* was a predominant regulator and controlled multiple altered pathways in ccRCC (regulated 36% significantly altered pathways of ccRCC) and PRCC (regulated 9% significantly altered pathways of PRCC) subtype, especially for ccRCC. *TNF* was also a predominant regulator and controlled multiple altered pathways in PRCC (regulated 9% significantly altered pathways of PRCC) and ChRCC (regulated 25% significantly altered pathways of ChRCC) subtype, especially for ChRCC. In addition, subtype-specific upstream regulators which affected a large number of perturbed pathways were also observed. *IFNG* and *TGFB1* for ccRCC (both of them regulated 36% significantly altered pathways of ccRCC), *SPP1, HDAC1* and *CUX1* (all of them regulated 9% significantly altered pathways of PRCC) for PRCC, and *STAT3, RB1, NUPR1* and *MECP2* (all of them regulated 8% significantly altered pathways of ChRCC) for ChRCC. Specifically, the recurrent upstream regulators presented distinct expression patterns among different subtypes of RCC (Fig. 5 and Table 2). Comparing with normal tissues, *TP63* was downregulated in ccRCC, PRCC and ChRCC; *TP53* was upregulated in ccRCC and PRCC; *TGFB1* was overexpressed in ccRCC and downexpressed in ChRCC. *IL15* was overexpressed in ccRCC and ChRCC; *TNF* was overexpressed in PRCC and downexpressed in ChRCC. Taken together, these results suggest that some of the altered pathways were regulated by several recurrent upstream regulators which were shared among distinct RCC subtypes, while presented different expression patterns. In addition, each subtype also has its own specific upstream regulators for a large number of perturbed pathways. Based on above results, we hypothesized that the various combination of a few common upstream regulators as well as a large number of subtype-specific upstream regulators work together to influence the downstream pathway perturbation of each subtype.

**Table 2:** The expression patterns of recurrent upstream regulators associated with multiply pathways

The potential upstream regulators	ccRCC	PRCC	ChRCC
TP63	−0.697	−2.514	−0.588
TP53	0.588	0.895	Ns
TGFB1	1.594	Ns	−0.638
IL15	0.9236	Ns	1.300
TNF	Ns	0.960	−0.876
IFNG	4.925	Ns	Ns
SPP1	Ns	1.563	Ns
STAT3	Ns	Ns	−1.145

Note: Values: log2FoldChange Value for each subtype compared with normal tissues; Ns: not significant differentially expressed comparing with normal tissues

### Identification of prognostic pathways

**Fig. 6:**
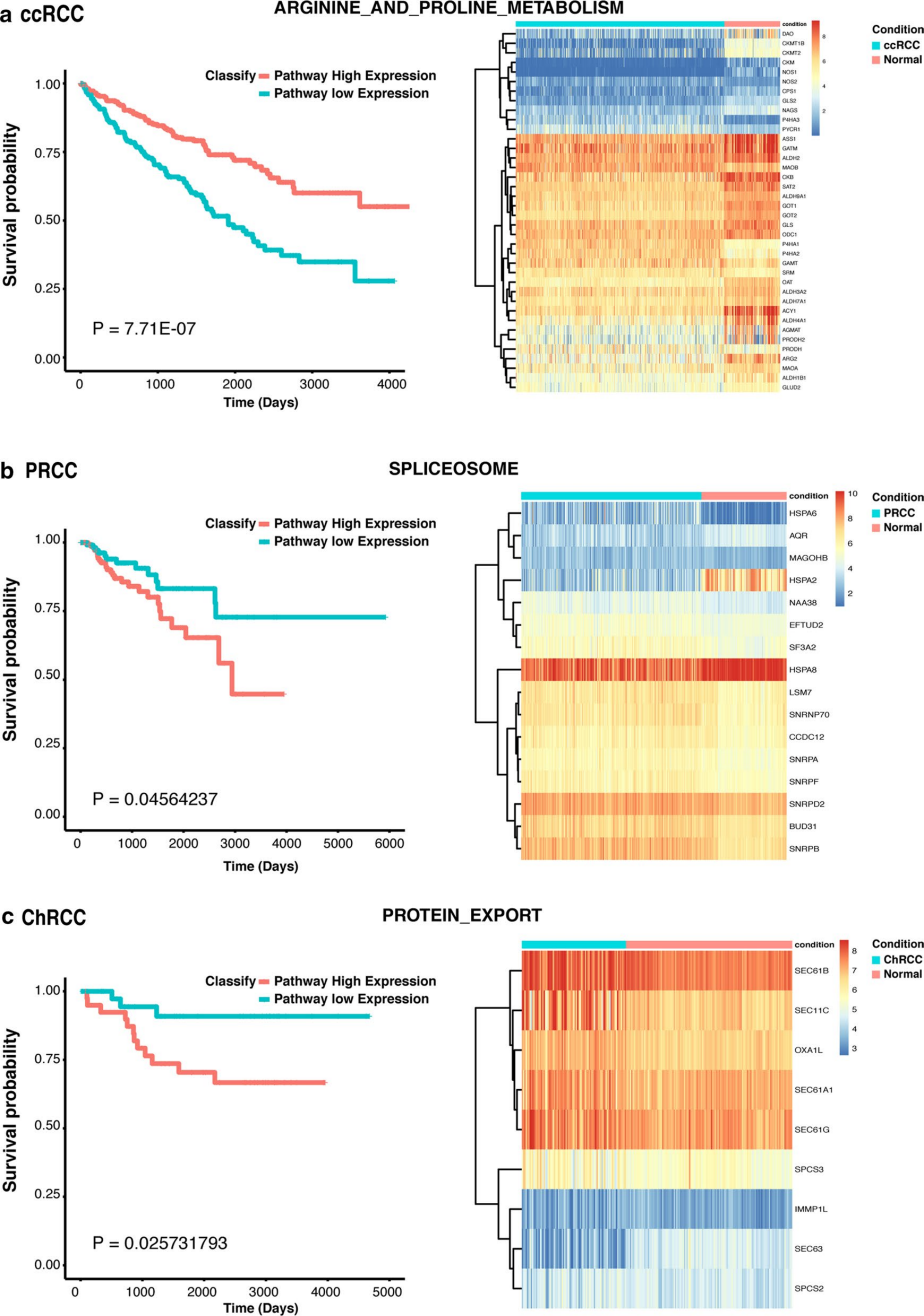
Identification of prognostic pathways based on pathway expression coupled with survival information for different subtypes of RCC. Left: Kaplan–Meier curves for prognostic pathways. Right: Heatmap of prognostic pathway among RCC subtypes and normal tissue based on the differentially expressed genes within the pathway. Blue indicates RCC subtype and red indicates normal tissue. **a** Example of a prognostic pathway in ccRCC; **b** Example of a prognostic pathway in PRCC; **c** Example of a prognostic pathway in ChRCC

Given the roles of activated pathways in cancer progression, we also investigated whether subtype-specific altered pathways were associated with patient survival within subtypes. As described in the Methods section, the pathway expression of each subtype was evaluated based on the expression of DEGs in a certain pathway. Then overall survival analysis was performed using the previously obtained pathway expression. A total of 23 pathways were selected as prognostic pathways (*P* < 0.05). ccRCC, PRCC and ChRCC contained 17, 3 and 3 prognostic pathways, respectively (Table 3). Examples of prognostic pathways of each subtype are shown in Fig. 6. After examining these survival-associated pathways, we found that all the prognostic pathways with lower expression associating with poor survival were observed in ccRCC. Moreover, all these pathways were also presenting lower expression compared with normal tissue, accounting 17 of 18 specific downregulated pathways (except for ENDOMETRIAL_CANCER) for ccRCC (Table 3). All the prognostic pathways with higher expression associating with poor survival were shown in PRCC and ChRCC. Particularly, for PRCC, the spliceosome pathway was upregulated, and vascular smooth muscle contraction and arrhythmogenic right ventricular cardiomyopathy were downregulated. For ChRCC, protein export was overexpressed, and axon guidance and small cell lung cancer were downregulated (Table 3).

**Table 3:** Prognostic pathways of various subtypes

Pathways	Log-rank test ***p***-value	Benjamini-Hochberg FDR q-value	Expression status associated with poor survival	Dysregulation against normal tissues	Subtype
PEROXISOME	6.30E-11	1.57E-09	Lower expression	Downregulated	ccRCC
FATTY_ACID_METABOLISM	3.17E-10	3.16E-09	Lower expression	Downregulated	ccRCC
ASCORBATE_AND_ALDARATE_METABOLISM	3.82E-10	3.16E-09	Lower expression	Downregulated	ccRCC
TERPENOID_BACKBONE_BIOSYNTHESIS	5.06E-10	3.16E-09	Lower expression	Downregulated	ccRCC
BETA_ALANINE_METABOLISM	7.84E-09	3.92E-08	Lower expression	Downregulated	ccRCC
DRUG_METABOLISM_CYTOCHROME_P450	3.78E-08	1.57E-07	Lower expression	Downregulated	ccRCC
BUTANOATE_METABOLISM	4.82E-08	1.72E-07	Lower expression	Downregulated	ccRCC
ARGININE_AND_PROLINE_METABOLISM	7.71E-07	2.41E-06	Lower expression	Downregulated	ccRCC
TIGHT_JUNCTION	6.81E-06	1.89E-05	Lower expression	Downregulated	ccRCC
SELENOAMINO_ACID_METABOLISM	1.14E-05	2.85E-05	Lower expression	Downregulated	ccRCC
METABOLISM_OF_XENOBIOTICS_BY_CYTOCHROME_P450	3.13E-05	7.11E-05	Lower expression	Downregulated	ccRCC
TYROSINE_METABOLISM	7.69E-05	0.000160213	Lower expression	Downregulated	ccRCC
PROSTATE_CANCER	0.001937092	0.003725177	Lower expression	Downregulated	ccRCC
CARDIAC_MUSCLE_CONTRACTION	0.003658082	0.006532289	Lower expression	Downregulated	ccRCC
EPITHELIAL_CELL_SIGNALING_IN_HELICOBACTER_PYLORI_INFECTION	0.010330511	0.017217519	Lower expression	Downregulated	ccRCC
FOLATE_BIOSYNTHESIS	0.021334521	0.033335189	Lower expression	Downregulated	ccRCC
GLUTATHIONE_METABOLISM	0.035577554	0.052319932	Lower expression	Downregulated	ccRCC
SMALL_CELL_LUNG_CANCER	0.014468144	0.102927174	Higher expression	Downregulated	ChRCC
AXON_GUIDANCE	0.021736945	0.102927174	Higher expression	Downregulated	ChRCC
PROTEIN_EXPORT	0.025731793	0.102927174	Higher expression	Upregulated	ChRCC
VASCULAR_SMOOTH_MUSCLE_CONTRACTION	0.0302394	0.14745595	Higher expression	Downregulated	PRCC
ARRHYTHMOGENIC_RIGHT_VENTRICULAR_CARDIOMYOPATHYARRHYTHMOGENIC_RIGHT_VENTRICULAR_CARDIOMYOPATHY	0.04406722	0.14745595	Higher expression	Downregulated	PRCC
SPLICEOSOME	0.04564237	0.14745595	Higher expression	Upregulated	PRCC

## Discussion

RCC consists of three major subtypes with different molecular characteristics and biological functions. Although lots of studies have addressed the molecular basis of various subtypes, the understanding of pathogenesis is still incomplete. Given the importance of pathways in charactering the biological processes and biological functions, we performed a pathway-based analysis to systematically explore the relationships among pathway perturbations, upstream regulators and associations with clinical outcome.

Based on the pathway-level analysis, pathway perturbations of various RCC subtypes have been identified compared with normal tissues. Specifically, both commonly altered pathways and subtype-specific altered pathways were obtained. Among the commonly altered pathways of RCC, proximal tubule bicarbonate reclamation has been highlighted, which is strongly associated with proximal tubule structure (proximal tubule is the originates of RCC) [19] and is consistent with the biological kidney function. In order to identify the distinctive characteristics of each subtype, we focused on the subtype-specific perturbed pathways. Among these subtype-specific altered pathways, we noted that many of them have been observed by others. Zeng et al. have highlighted that arginine and proline metabolism, butanoate metabolism, cardiac muscle contraction, fatty acid metabolism, selenoamino acid metabolism, tight junction were significantly downregulated pathways in ccRCC, and antigen processing and presentation, chemokine signaling pathway, natural killer cell mediated cytotoxicity were significantly upregulated pathways in ccRCC [20]. Perroud et al. also demonstrated that arginine and proline metabolism and butanoate metabolism were significantly downregulated in ccRCC with proteomics and metabolic profiling [21]. Xiao et al. have shown that ubiquitin mediated proteolysis was upregulated in ChRCC compared with normal tissue [22]. Tan et al. revealed that ERBB signaling pathway was upregulated in ChRCC [23]. All of these previous studies are consistent with our analysis results. However, for beta alanine metabolism and histidine metabolism pathway, our analysis show that the former is significantly disturbed in ccRCC, while the later one is significantly disturbed in ChRCC. These results partially disagree with recent work from Schaeffeler et al [24]. In their work, they showed that both pathways are significantly altered in ccRCC versus ChRCC [24]. The difference of histidine metabolism pathway may be due to the influence from downstream such as changing at proteomics levels, which result in the alterations of gene expressions that are not always consistent with the perturbations in metabolite levels. Taken together, we identified the significantly altered pathways of various RCC subtypes.

When we further investigate the potential upstream regulators of subtype-specific altered pathways, several recurrent regulators controlling multiple pathways were identified, such as *TP63, TP53*, *TGFB1, IFNG, IL1B* and *TNF*. Furthermore, a few of them are shared among ccRCC, PRCC and ChRCC, while presenting heterogeneous expression patterns among subtypes. In our study, *TP63* has been observed to play crucial roles in various RCC subtype and regulated multiply pathways. Tuna et al. have highlighted that *TP63* has a close relationship with tumor stage, grade and survival time of RCC [25]. Besides, we also observed that *TP53* as a critical tumor suppressor regulated various pathways in ccRCC and PRCC, and is overexpressed within each subtype compared against normal tissue. TP53 protein overexpression is particularly prevalent in RCC and is associated with higher tumor grades and poor survival [26]. Specifically, *TGFB1*, *IFNG, IL1B and TNF* are important proinflammatory cytokines, and have a close relationship with PD-L1 [27–30]*. TGFB1* is a key cytokine produced by proximal tubular and renal cancer cells, and regulates various vital processes and contributes to tumor progression and aggression in ccRCC [31, 32] . In our study, we found that *TGFB1* was one of the most active drivers, regulating about 36% perturbed pathways within ccRCC and showing higher expression compared with normal tissue. Ruan et al. have also shown that *TGFB1* is overexpressed in ccRCC against normal tissue [33]. *IFNG* is a major effector of immune therapy of cancer [34]*,* and its high expression is correlated with poor prognosis in ccRCC [35]*.* In addition, we have also observed that *IL1B* is upregulated in ccRCC. *IL1B* is produced by high malignancy ccRCC cells and its high expression has been shown to promote tumor aggressiveness [34, 35]. Taken together, we have identified the upstream regulators of various RCC subtypes, including both common and subtype-specific upstream regulators. Some of the recurrent upstream regulators controlling a few of the altered pathways were shared among distinct RCC subtypes, which presented different expression patterns. The recurrent regulators as well as subtype-specific regulators work together to promote tumor progression. As some of the recurrent regulators are also proinflammatory cytokines, blocking such kind of immune checkpoints would provide promising potential for personalized therapeutic intervention.

Since pathway perturbation plays vital roles for cancer progression, we also identified prognostically disturbed pathways. Among these pathway predictors, we found that pathways with lower expression linked to poor outcome were presented in ccRCC. Meanwhile, we also observed that these prognostic pathways exhibited downregulation in ccRCC. Thus, we hypothesize that the alteration of upstream regulators induced the downregulation of such kind of prognostic pathways, thus contributing to the tumorigenesis and progression of ccRCC. Instead, pathways with higher expression linked to poor outcome were present in PRCC and ChRCC. Among these pathway predictors, we noticed that spliceosome and protein export were upregulated in PRCC and ChRCC compared to normal tissue, respectively. Given that frequent splicing and export of protein are necessary events for tumor proliferation, we hypothesize that the overexpression of spliceosome and protein export functions promote tumorigenesis and progression of PRCC and ChRCC, respectively. As for vascular smooth muscle contraction and arrhythmogenic right ventricular cardiomyopathy both of them were downregulated in PRCC compared to normal tissue. Considering the highly vascularized nature of the kidney tissue, we hypothesize that these perturbations reflect a disruption in the biological and physiological functions of the kidney.

Despite the extensive observations and results generated from our analysis, some limitations of this study should be noticed as well. First, the mRNA levels of pathway may not represent all the biological function due to lack of large-scale downstream information, such as protein expression levels and modulation (for example, acetylation and phosphorylation) and metabolite abundance. Secondly, there are complex interactions between pathways and they often function together to contribute tumorigenesis and progression. Therefore, pathway perturbation analysis based on individual pathways may only estimate a portion of its activity. Thirdly, while the significantly altered pathways of various subtypes have been defined, further research is needed to explain the underly mechanistic meaning and implications of the altered pathway in the tumorigenic process of RCCs. Last but not least, even though the potential upstream regulators of a certain pathway have been identified and their roles in various subtypes have been confirmed in many recent studies, further confirmation of how changes in upstream regulators affect pathway alterations still requires experimental validation.

## Conclusions

In summary, we performed a pathway-based analysis among different RCC subtypes, and both the commonly altered pathways and subtype-specific altered pathways were identified. We found that some of the recurrent upstream regulators controlled a few of the altered pathways and exhibited different expression patterns among various RCC subtypes. Each subtype also has its own specific upstream regulators for a large number of perturbed pathways. In addition, prognostic pathways were also identified. We hypothesized that the dysregulation of recurrent upstream regulators as well as subtype-specific upstream regulators work together to affect pathway perturbations and further influence cancer prognosis among various RCC subtypes. Our findings can catalyze a better understanding of the mechanisms of various RCC subtypes and provide promising potential targets for personalized therapeutic intervention.

## Supplementary Information

**Additional file 1: Table S1.** Differentially expressed genes within pathways.

**Additional file 2: Table S2.** Significantly altered pathways of various subtypes.

**Additional file 3: Table S3.** Commonly altered pathways and subtype-specific altered pathways.

**Additional file 4: Table S4.** Overlapped genes in common altered pathways among ccRCC, PRCC and ChRCC.

**Additional file 5: Table S5.** Potential upstream regulators.

**Additional file 6: Figure S1.** An example of the correlations between upstream regulators with the target genes.

## Data Availability

The TCGA data used in this study are available in Broad GDAC Firehose (https://gdac.broadinstitute.org). The re-evaluated histological subtypes of kidney cancer samples are available from Ricketts et al. [1]. The authors declare that all the other data supporting the findings of this study are available within the article and its supplementary information files and from the corresponding author on reasonable request.

## Abbreviations

RCC: Renal cell carcinoma
ccRCC: Clear cell renal cell carcinoma
PRCC: Papillary renal cell carcinoma
ChRCC: Chromophobe renal cell carcinoma
DEGs: differentially expressed genes
