# Supplementary figures and images for: A pan-kidney cancer study identifies subtype specific perturbations on pathways with potential drivers in renal cell carcinoma
Source: BMC Med Genomics. 2020 Dec 28;13(Suppl 11):190. doi: 10.1186/s12920-020-00827-5 (PMC7771093; doi:10.1186/s12920-020-00827-5)

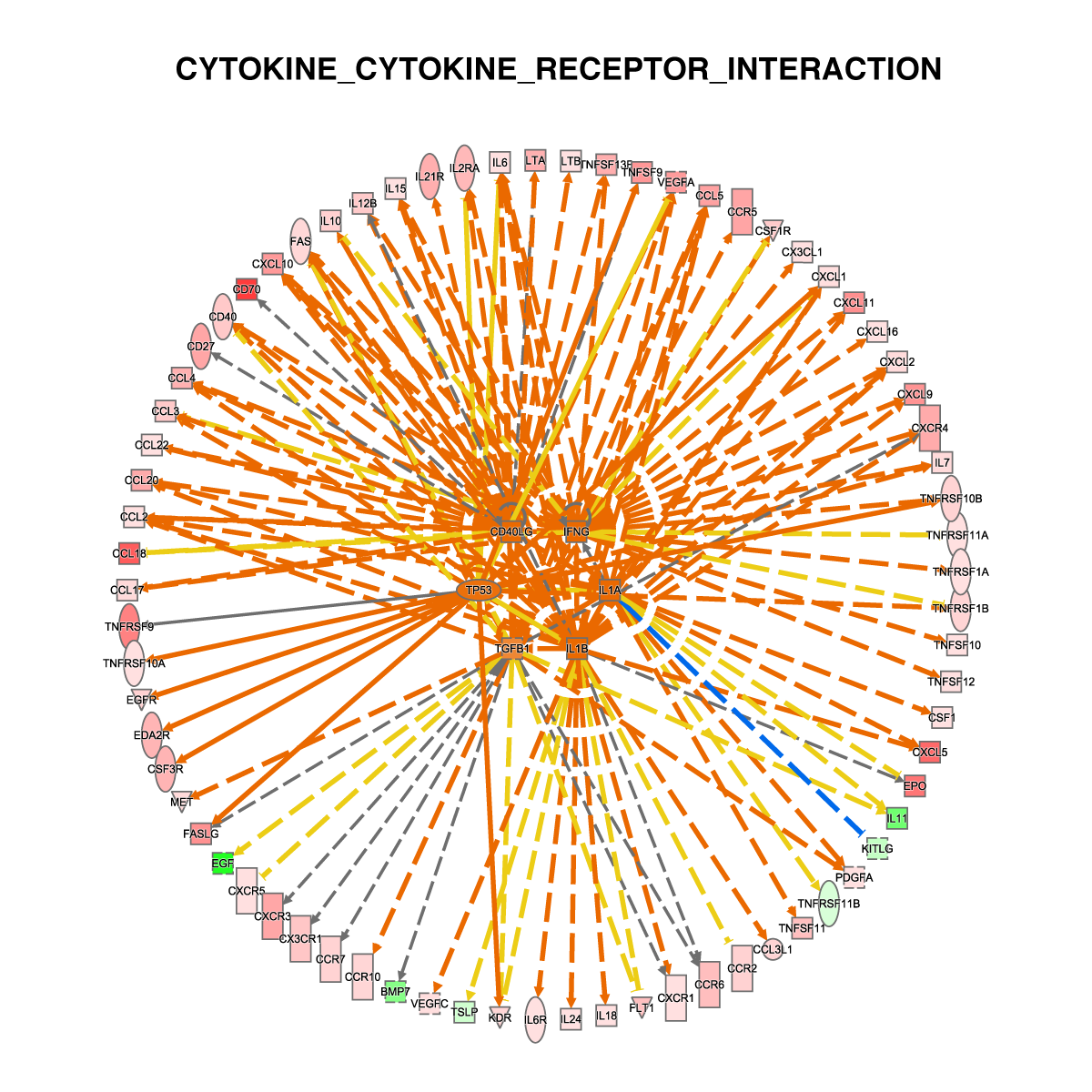

Supplement: Supplementary file 6 — Additional file 6: Figure S1. An example of the correlations between upstream regulators with the target genes. [file 12920_2020_827_MOESM6_ESM.tif]
